# Longitudinal Temporal Mediation Within the Motivational Phase of the Integrated Motivational‐Volitional (IMV) Model of Suicidal Behavior With Moderation of Loneliness and Resilience

**DOI:** 10.1111/sltb.70081

**Published:** 2026-02-22

**Authors:** Jiarui Xiao, Karen Wetherall, Seonaid Cleare, Mareike Ernst, Kathryn A. Robb, Rory C. O'Connor

**Affiliations:** ^1^ Suicidal Behaviour Research Laboratory, School of Health and Wellbeing University of Glasgow Glasgow UK; ^2^ Department of Clinical Psychology, Psychotherapy and Psychoanalysis, Institute of Psychology University of Klagenfurt Klagenfurt am Wörthersee Austria

**Keywords:** defeat, entrapment, integrated motivational‐volitional (IMV) model, loneliness, resilience, suicidal ideation

## Abstract

**Background:**

The Integrated Motivational‐Volitional (IMV) Model of Suicidal Behavior places defeat and entrapment central to the development of suicidal ideation. Factors such as loneliness and resilience may moderate risk. This study examines the longitudinal relationship between these variables.

**Methods:**

Secondary data from the UK COVID‐19 Mental Health and Wellbeing Study (COVID‐MH) were analyzed (*n* = 2518). Defeat, entrapment, loneliness, resilience, and suicidal ideation were measured across 3 waves (March to May 2020). Six longitudinal mediation models were tested, each with wave 2 entrapment (or subscales) as mediator of wave 1 defeat and wave 3 suicidal ideation. Loneliness and resilience (from waves 1 and 2) were included as moderators of the associations between defeat and entrapment with suicidal ideation.

**Results:**

Wave 2 entrapment mediated the relationship between wave 1 defeat and wave 3 suicidal ideation, controlling for wave 1 entrapment and suicidal ideation. Wave 1 loneliness and resilience moderated the pathway from defeat to entrapment, but wave 2 resilience or loneliness did not moderate the pathway from entrapment to suicidal ideation.

**Conclusion:**

Findings provide robust longitudinal support for the motivational phase of the IMV model and highlight the importance of targeting entrapment and loneliness and enhancing resilience.

## 1 Introduction

Suicide prevention is a major public health priority and this is even more urgent with suicide rates increasing in recent years in many Western countries (World Health Organization 2025). As a result, early recognition of the signs of suicide risk and timely suicide prevention efforts such as psychological support interventions are critical. Such interventions can significantly improve an individual's mental health, reduce the risk of suicide, and ultimately help to protect lives (Singh et al. 2022). Suicidal ideation, in which an individual experiences thoughts of ending their own life, which may be fleeting thoughts or elaborate plans, is an important warning sign and opportunity for intervention in suicide prevention (Harmer et al. 2024). Improved identification and intervention for suicidal ideation is a key strategy for reducing suicide attempts and related deaths (Jobes and Joiner 2019). Therefore, exploring how suicidal ideation develops can help inform more effective intervention strategies and improve suicide prevention outcomes.

### 1.1 Understanding the Emergence of Suicidal Ideation

**FIGURE 1:**
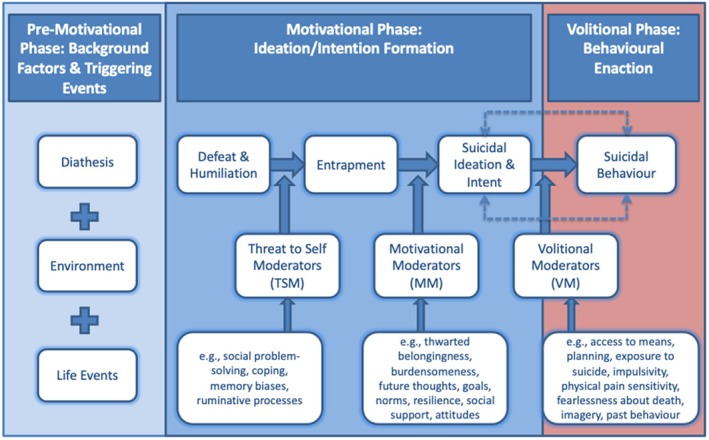
The IMV model of suicidal behavior (O'Connor and Kirtley 2018).

To aid our understanding of how suicidal ideation and behaviour emerge and are enacted, frameworks incorporating a range of biopsychosocial risk and protective factors for suicide have been developed. Such models highlight the different factors that may precede suicide risk, such as feeling hopeless, trapped, or a burden (Joiner 2005; Klonsky and May 2015; O'Connor and Kirtley 2018). One predominant model is the Integrated Motivational‐Volitional Model of Suicidal Behavior (IMV; Figure 1) that suggests suicidal ideation stems from an unbreakable sense of defeat (feeling that one has failed and lost one's place), leading to the individual feeling trapped (entrapment) in a painful and powerless situation from which they cannot escape (O'Connor and Kirtley 2018). The IMV model suggests that suicidal behaviours emerge across three phases: the pre‐motivational phase (the background environment that influences suicide risk, such as poverty, genetics, and negative life events), the motivational phase (the factors that contribute to the formation of suicidal ideation, such as defeat and entrapment), and the volitional phase (the factors that contribute to the progression from suicidal ideation to behaviour, such as exposure to suicide and ready access to the means of suicide) (O'Connor 2011).

Evidence supporting the IMV model's motivational phase is growing, whereby defeat leads to entrapment, which further triggers and intensifies the formation of suicidal ideation (Wetherall, Cleare, Eschle, et al. 2022; Ballegooijen et al. 2022). Specifically, individuals experience a sense of defeat (e.g., failure) following challenging life events or feelings of shame (Gilbert and Allan 1998). This can lead to feelings of entrapment, with individuals feeling they cannot escape from challenging situations or pressures, including feeling trapped by external (e.g., relationships) and internal (e.g., negative thoughts) factors (Owen et al. 2018; O'Connor and Kirtley 2018). The IMV model proposes entrapment is the proximal predictor of suicidal ideation emerging, and several studies have supported this (e.g., Wetherall, Cleare, McClelland, et al. 2022; Dhingra et al. 2016; Rasmussen et al. 2010), with internal entrapment potentially being more harmful (Wetherall, Cleare, McClelland, et al. 2022). However, much of the mediation research conducted testing the motivational phase of the IMV (i.e., defeat leads to entrapment, entrapment leads to suicidal ideation) has been limited to cross‐sectional designs (e.g., Rasmussen et al. 2010) or has utilized only two time points (e.g., Wetherall, Cleare, McClelland, et al. 2022). Inferring causality through mediation analysis assumes temporality, specifically, that the exposure precedes the mediator, and the mediator precedes the outcome (Narita et al. 2025). Therefore, this will be the first analysis to truly test the causality of the motivation phase of the IMV model using three time points of data, and controlling for baseline measures throughout, as suggested by Narita et al. (2025).

In addition, the IMV model describes several factors that may moderate the pathways (see Figure 1), accelerating or decelerating the process of defeat leading to entrapment or suicidal ideation (Wetherall et al. 2018). In this study, we have focused on two such moderators, namely resilience and loneliness, as they are established correlates of suicidal ideation (McClelland et al. 2021; Gooding et al. 2015). Resilience refers to an individual's ability to adapt and recover in the face of negative events such as adversity and stress (Fletcher and Sarkar 2013). Evidence suggests resilience moderates the relationship between defeat, entrapment, and hopelessness; the higher the resilience, the lower the risk of suicide (Gooding et al. 2015). This could indicate that, within the context of the IMV model, resilience can safeguard against motivational escalation by reducing the likelihood that defeat and entrapment translate into suicidal ideation. However, to our knowledge, this moderating influence has not been tested longitudinally across each IMV pathway, which constitutes a research gap the present study aims to address.

Loneliness refers to an individual's perceived lack of meaningful social connection, both in a quantitative and qualitative way (Perlman and Peplau 1981). It is conceptually similar to thwarted belongingness, a central social‐cognitive vulnerability factor in the Interpersonal Theory of Suicide (Joiner et al. 2010). It was associated with higher suicidal ideation at the within‐person level in ambulatory assessment studies, which underlines that it contributes to a proximal, context‐sensitive risk process rather than merely being a stable correlate of suicidal ideation (Ernst et al. 2025). Loneliness has been identified as a moderating factor on the pathway between defeat and entrapment, as well as between entrapment and self‐harm ideation (McClelland et al. 2021). Its conceptual role as a socially embedded “threat‐to‐self” cue makes loneliness particularly relevant when examining how individuals interpret and respond to internal states of defeat. Taken together, loneliness and resilience offer theoretically grounded and mechanistically complementary moderating influences: loneliness as a social threat amplifier and resilience as a protective psychological resource.

As there is evidence of potential moderation effects of these factors on both the defeat to entrapment and the entrapment to suicidal ideation pathways, the current study will test moderation effects upon both pathways. This allows us to investigate not simply whether suicidal ideation arises from defeat and entrapment, but under which psychosocial conditions these processes are most likely to unfold.

### 1.2 Current Study

A growing number of studies support the IMV model of suicidal behavior. However, there is still a need for longitudinal studies using data collected at three different time points to accurately test the temporal relationship assumed by mediation analysis. Therefore, in the current study, we used data from the first three waves of the representative UK COVID‐19 Mental Health and Wellbeing Study (COVID‐MH), which included the measurement of the core variables of the motivational phase of the IMV model (i.e., defeat, entrapment and suicidal ideation) and potential moderators (i.e., loneliness and resilience). The aims of the current study were (1) to examine whether entrapment mediates the relationship between defeat and suicidal ideation longitudinally and (2) to explore the moderating role of loneliness and resilience in the pathways from defeat to entrapment, and from entrapment to suicidal ideation.

## 2 Method

### 2.1 Participants and Procedure

The study used a longitudinal study design with adults from across the UK; the non‐probability sample was collected during the early stages of the COVID‐19 pandemic (March 2020) with quota sampling based on gender, age, region, and socioeconomic group. The participant characteristics are shown in Table 1 (*n* = 2518). Due to the constraints of home isolation during the pandemic, the choice of quota sampling for online recruitment helped to collect data from a stratified UK sample. For more details about the study recruitment and measures, please see O'Connor et al. (2021) and Wetherall, Cleare, Eschle, et al. (2022).

**TABLE 1:** Demographic characteristics of participants (*n* = 2518).

Characteristic	Total: *n* (%)
Sex at birth	
Male	1171 (46.5)
Female	1344 (53.4)
Prefer not to answer	3 (0.1)
Age	
18–29 years	592 (23.5)
30–59 years	1386 (55.0)
> 60 years	540 (21.5)
Ethnicity	
White	2310 (91.7)
Asian	70 (2.8)
Black	68 (2.8)
Mixed	52 (2.0)
Other/Unknown	18 (0.7)
Region of UK	
South England	1043 (41.4)
North England	687 (27.3)
Midlands	333 (13.2)
Scotland	298 (11.8)
Wales	105 (4.2)
Northern Ireland	52 (2.1)
Socioeconomic group	
High	1442 (57.3)
Low	1076 (42.7)
Employment status	
Employed	1484 (58.9)
Unemployed	298 (11.8)
Other (retired, education, homemaker)	736 (29.3)

Specifically, between 31 March to 9 April 2020 (wave 1), UK online panel (Panelbase.net) members were invited by email to participate in an online survey on mental health and wellbeing. Respondents answered demographic questions to determine eligibility based on predefined quotas, these quotas were established according to key demographic variables such as age, gender, UK region, and socioeconomic group. The target sample was 3000 and the final sample was 3077. All participants in wave 1 of the survey were invited to participate in subsequent waves, and those who did not participate in wave 2 were not excluded from wave 3. In wave 2 (10 April to 27 April 2020), 89% (*n* = 2742) of participants completed the survey; in wave 3 (28 April to 11 May 2020), 85% (*n* = 2604) of participants completed the survey. A total of 2518 participants completed all three waves of the survey and were included in the statistical analyses. There were 8 waves in total, with data collection continuing until June 2021. Measures across the waves included questions regarding participants demographics, experiences during the COVID‐19 pandemic, physical health, mental health (i.e., depression, anxiety), and key psychosocial measures (i.e., loneliness, worry, wellbeing). Repeated measures of defeat, entrapment and suicidal ideation allowed for a longitudinal test of the IMV model. In the current analysis, we opted to focus on longitudinal data from the first three waves as evidence suggests that shorter follow‐up periods may be better suited to account for the fluctuations in psychological variables and suicidal thinking (e.g., Coppersmith et al. 2023).

A test of demographic differences between participants who completed the full survey and those who dropped out found significant differences (*p* < 0.05) in age, gender, and initial suicidal ideation (wave 1). The results indicated that the dropout rate was 30.1% in the 18–34 age group, 17.9% in the 35–54 age group, and 8.7% in the 55 and over age group. In addition, male participants had a significantly lower dropout rate (6.8%) than female participants (11.3%). Finally, the presence of suicidal ideation was significantly associated with higher study dropout rates.

Ethical approval was obtained from the University of Glasgow's Medical, Veterinary, and Life Sciences Ethics Committee (approval number: 200190146) and the study was conducted in accordance with the Declaration of Helsinki (World Medical Association 2013). Participants provided informed consent prior to participating and could withdraw at any time. The study was pre‐registered at aspredicted.org (#41910). Participants were rewarded £1.50 for each survey completed and received information about available mental health support organizations (O'Connor et al. 2021).

### 2.2 Measures

#### 2.2.1 Demographics

Participant demographics were recorded at the beginning of the survey. This included age, sex, ethnicity, region of the UK, socioeconomic group, and employment status. Participant demographics are displayed in Table 1.

#### 2.2.2 Defeat

Defeat was assessed using four items from the defeat subscale of Griffiths' short‐form scale (e.g., “I feel that I am one of life's losers”), which demonstrated high internal consistency (*α* = 0.88 to 0.94) (Griffiths et al. 2015). There are five response options for each item, ranging from “Never” to “Always,” reflecting the severity of the feeling of defeat, with higher scores indicating high levels of feelings of defeat.

#### 2.2.3 Entrapment

Entrapment was measured using the four‐item Brief Entrapment Scale (De Beurs et al. 2020), which exhibited a strong internal consistency with Cronbach's alpha of 0.87. Each item has five response options, ranging from “Not at all like me” to “Extremely like me,” with higher levels indicating greater feelings of entrapment. The measure includes subscale if external (i.e., life circumstances you wish to escape from) and internal (i.e., negative thoughts and feelings) entrapment, with two items assessing external entrapment (“I often have the feeling that I would just like to run away”, “I feel powerless to change things”) and two items for internal entrapment (“I feel trapped inside myself”, “I feel I'm in a deep hole I can't get out of”). The subscales of the entrapment scale will be used within a sensitivity analysis.

#### 2.2.4 Loneliness

Loneliness was measured using a three‐item scale derived from the Revised UCLA Loneliness Scale (Hughes et al. 2004), which showed excellent internal consistency, as evidenced by a Cronbach's alpha of 0.91 (e.g., “How often do you feel isolated from others?”). Each item offers three response options, ranging from “Hardly ever” to “Often,” with higher frequencies indicating greater loneliness.

#### 2.2.5 Resilience

Resilience was measured using four items from the Brief Resilience Scale (Campbell‐Sills and Stein 2007), which demonstrated strong internal consistency with a Cronbach's alpha of 0.85 (e.g., “Can deal with whatever comes”). Each item has five response options, extending from 0 (“Not true at all”) to 4 (“True almost always”), with higher scores reflecting higher levels of resilience.

#### 2.2.6 Suicidal Ideation

Suicidal ideation was measured with a question that included frequency over the past week, as suggested by common measures of suicidality (e.g., Columbia Suicide Severity Rating Scale (C‐SSRS); Posner et al. 2011), as this would aid replicability across the shorter waves of the study. Participants were asked: “In the past week, how often have you thought about suicide?” Response options included “one day,” “a few days,” “more than half of the days,” “almost every day,” “never,” and “I'd rather not answer.” Responses varying from “one day” to “almost every day” were classified as “yes,” while “never” was categorized as “no”. Those who did not answer were treated as missing data.

### 2.3 Statistical Analysis

Data analysis was conducted with the IBM SPSS version 29. As only suicidal ideation had missing data, it was decided to use listwise deletion. Initially, correlation analyses were conducted to test the relationships between all variables included in the study. The PROCESS macro tool (Hayes 2017) for SPSS was used to conduct the mediation (PROCESS Model 4) and moderated‐mediation (PROCESS Models 7 and 14) analyses. Specifically, we conducted a mediation analysis (models 1 and 2) testing the indirect effects of wave 2 entrapment on the relationship between wave 1 defeat and wave 3 suicidal ideation, controlling for wave 1 entrapment and suicidal ideation. As internal entrapment has been shown to be particularly pernicious for suicide risk, we ran a sensitivity analysis with wave 2 internal and external entrapment as parallel mediators in the relationship between wave 1 defeat and wave 3 suicidal ideation.

A further 4 moderated mediation models were conducted that included the testing of moderators at different stages of the longitudinal pathways of Model 1. Specifically, models included the moderation of wave 1 loneliness (model 3) and wave 1 resilience (model 5) on the path from wave 1 defeat to wave 2 entrapment. The next models included the moderation of wave 2 loneliness (model 4) and wave 2 resilience (model 6) on the path from wave 2 entrapment to wave 3 suicidal ideation. The PROCESS macro performs a simple slopes analysis as part of the moderation analysis to show how the relationship between the variables changes as a function of the moderator variable, by indicating the size of the relationship at low (sample‐level mean—1SD), medium (mean) and high (mean + 1 SD) levels of the moderator. To enhance the accuracy of the estimated confidence intervals for the effects (Hayes 2017), we performed 10,000 bootstrap procedures. All models controlled for wave 1 entrapment and wave 1 suicidal ideation.

## 3 Results

### 3.1 Correlation Analysis

Before proceeding to the path analyses, partial correlations are explored (see the top half of Table 2). When controlling for wave 1 entrapment and suicidal ideation, all the study variables remained significantly correlated with wave 3 suicidal ideation, although the effect sizes were smaller.

**TABLE 2:** Correlations, partial correlations, and means (SD) of all study variables (*n* = 2518).

Variable	Wave 1 defeat	Wave 1 entrapment	Wave 1 suicidal ideation	Wave 1 resilience	Wave 1 loneliness	Wave 2 entrapment	Wave 2 resilience	Wave 2 loneliness	Wave 3 suicidal ideation
Wave 1 defeat	—	—	—	−0.26***	0.25***	0.25***	−0.25***	0.25***	0.04*
Wave 1 entrapment	0.84***	—	—	—	—	—	—	—	—
Wave 1 suicidal ideation	0.39***	0.41***	—	—	—	—	—	—	—
Wave 1 resilience	−0.54***	−0.50***	−0.23***		−0.21***	−0.17***	0.67***	−0.19***	−0.07***
Wave 1 loneliness	0.58***	0.56***	0.26***	−0.43***		0.18***	−0.22***	0.70***	0.04*
Wave 2 entrapment	0.76***	0.80***	0.38***	−0.49***	0.54***		−0.26***	0.32***	0.17***
Wave 2 resilience	−0.50***	−0.46***	−0.23***	0.74***	−0.42***	−0.51***		−0.24***	−0.08***
Wave 2 loneliness	0.54***	0.51***	0.24***	−0.40***	0.78***	0.57***	−0.42***		0.05*
Wave 3 suicidal ideation	0.34***	0.35***	0.53***	−0.23***	0.24***	0.39***	−0.24***	0.23***	
Mean (SD)	4.11 (3.88)	3.80 (4.33)	0.08 (0.26)	14.3 (3.96)	5.15 (1.91)	3.67 (4.20)	14.31 (3.78)	5.14 (1.90)	0.10 (0.30)

*Note:* Correlations are reported on the bottom half of the table. Partial correlations are reported on the top‐half of the table.

* *p* < 0.05.

*** *p* < 0.001.

### 3.2 Testing the Pathways From Defeat to Entrapment to Suicidal Ideation

#### 3.2.1 Model 1: Entrapment as a Mediator of the Defeat to Suicidal Ideation Pathway

**FIGURE 2:**
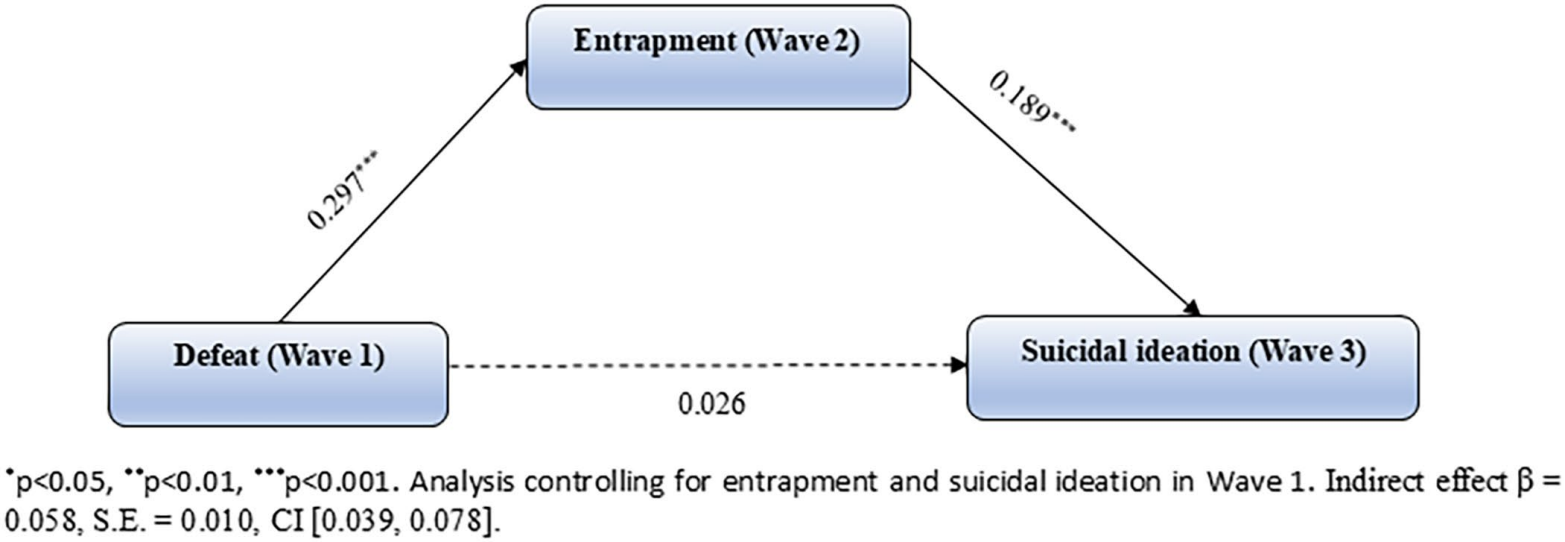
Model 1: mediation model with entrapment mediating the relationship between defeat and suicidal ideation (*n* = 2401). ***p < 0.001. Analysis controlling for entrapment and suicidal ideation in Wave 1. Indirect effect *β* = 0.058, SE = 0.010, CI [0.039, 0.078].

The mediation model demonstrated that entrapment mediated the pathway from defeat to suicidal ideation longitudinally. As shown in Figure 2, wave 1 defeat significantly predicted wave 2 entrapment (*β* = 0.297, SE = 0.024, CI [0.250, 0.343], *p* < 0.001), and wave 2 entrapment in turn significantly predicted wave 3 suicidal ideation (*β* = 0.189, SE = 0.028, CI [0.134, 0.244], *p* < 0.001). The indirect effect of entrapment upon the relationship between defeat and suicidal ideation was significant (*β* = 0.058, SE = 0.010, CI [0.039, 0.078]). Further, the direct effect of defeat on suicidal ideation was not statistically significant (*β* = 0.026, SE = 0.038, CI [−0.047, 0.100], *p* = 0.484).

#### 3.2.2 Model 2: Internal and External Entrapment as Parallel Mediators of the Defeat to Suicidal Ideation Pathway

As a sensitivity analysis, we ran the mediation model with wave 2 internal and external entrapment as parallel mediators of the relationship between wave 1 defeat and wave 3 suicidal ideation. This model is included within the supplemental materials (Figure S1; Section S1). We found that the indirect effect of internal entrapment (*β* = 0.037, SE = 0.011, CI [0.018, 0.059]), but not external entrapment (*β* = 0.014, SE = 0.011, CI [−0.008, 0.037]) was statistically significant, suggesting that only internal entrapment mediated the relationship between defeat and subsequent suicidal ideation. However, as the effect of total entrapment was stronger (*β* = 0.058), we decided to complete the moderated mediated analysis with the total entrapment measure. Additionally, further sensitivity analysis found that the moderator effects were replicated exactly with internal entrapment as the mediator (Table S1).

#### 3.2.3 Model 3: Loneliness as a Moderator of the Defeat to Entrapment Pathway

**FIGURE 3:**
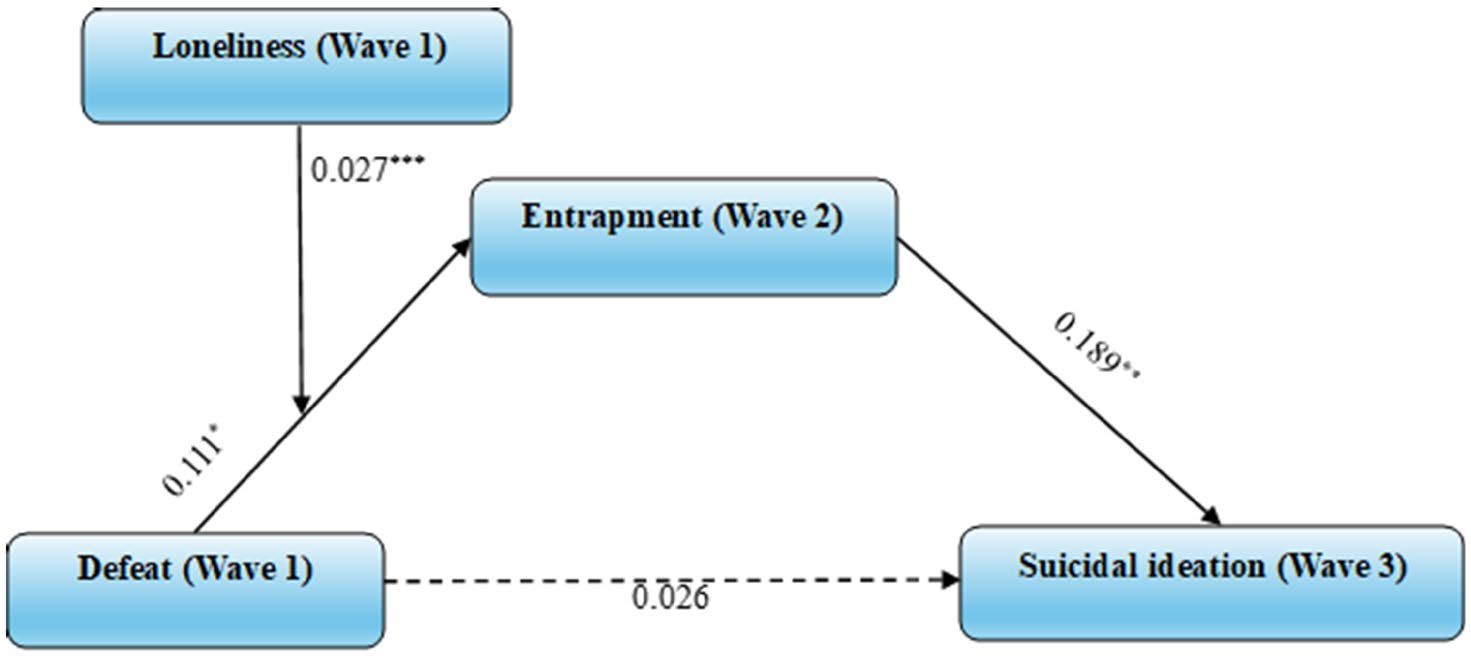
Model 3: moderated mediation model of entrapment and loneliness between defeat and suicidal ideation (*n* = 2401). *p < 0.05, **p < 0.01, ***p < 0.001. Analysis controlling for entrapment and suicidal ideation in Wave 1.

The first moderated mediation model tested the moderating role of wave 1 loneliness in the pathway by which wave 1 defeat affects wave 3 suicidal ideation via wave 2 entrapment. As shown in Figure 3, wave 1 defeat significantly predicted wave 2 entrapment (*β* = 0.111, SE = 0.045, CI [0.024, 0.199], *p* = 0.013), and wave 2 entrapment in turn significantly predicted wave 3 suicidal ideation (*β* = 0.189, SE = 0.028, CI [0.134, 0.244], *p* < 0.001). Further, the direct effect of defeat on suicidal ideation was not statistically significant (*β* = 0.026, SE = 0.038, CI [−0.047, 0.100], *p* = 0.484). The interaction between defeat and loneliness was significant (*β* = 0.027, SE = 0.007, CI [0.013, 0.040], *p* < 0.001), indicating that loneliness moderates the pathway between defeat and entrapment.

Indirect effects of defeat on suicidal ideation through entrapment yielded the following outcomes: when loneliness was low, the effect size was 0.036, SE = 0.008, 95% CI [0.021, 0.054]; for average loneliness, the effect size was 0.046, SE = 0.009, 95% CI [0.030, 0.065]; and for high loneliness, the effect size was 0.056, SE = 0.011, 95% CI [0.036, 0.079]. Furthermore, suicidal ideation (wave 1) (*β* = 2.788, SE = 0.213, CI [2.370, 3.206], *p* < 0.001) significantly predicted suicidal ideation (wave 3) in the model, whereas entrapment (wave 1) (*β* = −0.024, SE = 0.035, CI [−0.091, 0.044], *p* = 0.497) had non‐significant main effects.

#### 3.2.4 Model 4: Loneliness as a Moderator of the Entrapment to Suicidal Ideation Pathway

Model 4 examined whether wave 2 loneliness moderated the pathway from wave 2 entrapment to wave 3 suicidal ideation. Results did not support a moderating effect of wave 2 loneliness on this pathway. Specifically, the interaction between wave 2 entrapment and wave 2 loneliness was non‐significant (*β* = −0.004, SE = 0.009, CI [−0.022, 0.015], *p* = 0.696), although the indirect effect of wave 1 defeat on wave 3 suicidal ideation through wave 2 entrapment remained significant (Figure S2). Thus, while mediation was observed, the evidence did not support the moderating effect of loneliness on the relationship between entrapment and suicidal ideation (see Supporting Information Section S2).

#### 3.2.5 Model 5: Resilience as a Moderator of the Defeat to Entrapment Pathway

**FIGURE 4:**
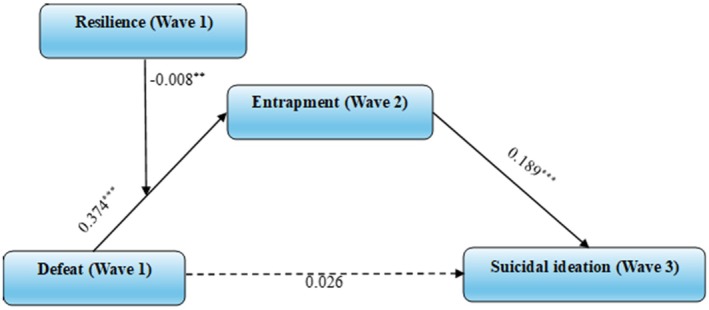
Model 5: moderated mediation model of entrapment and resilience between defeat and suicidal ideation (*n* = 2401). **p < 0.01, ***p < 0.001. Analysis controlling for entrapment and suicidal ideation in Wave 1.

Model 5 tested the moderating role of wave 1 resilience in the pathway by which wave 1 defeat affects wave 3 suicidal ideation via wave 2 entrapment. As shown in Figure 4, wave 1 defeat significantly predicted wave 2 entrapment (*β* = 0.374, SE = 0.048, CI [0.281, 0.467], *p* < 0.001), and wave 2 entrapment in turn significantly predicted wave 3 suicidal ideation (*β* = 0.189, SE = 0.028, CI [0.134, 0.244], *p* < 0.001). Further, the direct effect of defeat on suicidal ideation was not statistically significant (*β* = 0.026, SE = 0.038, CI [−0.047, 0.100], *p* = 0.484). The interaction between wave 1 defeat and wave 1 resilience was significant (*β* = −0.008, SE = 0.003, CI [−0.014, −0.002], *p* < 0.01), indicating that resilience moderates the pathway between defeat and entrapment.

Indirect effects of defeat on suicidal ideation through entrapment yielded the following outcomes: when resilience was low, the effect size was 0.053, SE = 0.010, 95% CI [0.035, 0.075]; for average resilience (the 50th percentile), the effect size was 0.047, SE = 0.009, 95% CI [0.030, 0.067]; and for high resilience, the effect size was 0.040, SE = 0.010, 95% CI [0.024, 0.061]. As with model 1, suicidal ideation (wave 1) (*β* = 2.788, SE = 0.213, CI [2.370, 3.206], *p* < 0.001) significantly predicted suicidal ideation (wave 3) in the model, whereas entrapment (wave 1) (*β* = −0.024, SE = 0.035, CI [−0.091, 0.044], *p* = 0.497) had non‐significant main effects.

#### 3.2.6 Model 6: Resilience as a Moderator of the Defeat to Suicidal Ideation Pathway

The final moderated mediation model (Model 6) examined whether wave 2 resilience moderated the pathway from wave 2 entrapment to wave 3 suicidal ideation. Results did not support a moderating effect of resilience on this pathway. Specifically, the interaction between wave 2 entrapment and wave 2 resilience was non‐significant (*β* = 0.001, SE = 0.004, CI [−0.007, 0.009], *p* = 0.798), although the indirect effect of wave 1 defeat on wave 3 suicidal ideation through wave 2 entrapment remained significant (Figure S3, Supporting Information Section S3).

## 4 Discussion

The current study provides a true longitudinal temporal test of the Integrated Motivational Volitional (IMV) model of suicidal behavior using measures of the core components of the motivational phase of the model. Specifically, across three time points spanning six weeks, the causal relationship between defeat, entrapment, and suicidal ideation was tested, controlling for baseline measures to adjust for confounding effects. The moderating role of loneliness and resilience in the defeat‐entrapment‐suicidal ideation pathway was also tested. For the first study aim, the findings supported the IMV model's central premise that the causal relationship between defeat and suicidal ideation is mediated by entrapment, with all path models tested finding support for this indirect relationship longitudinally. For the second study aim, loneliness and resilience were found to moderate the defeat‐to‐entrapment pathway only, suggesting that those high in loneliness are more likely to experience entrapment, and those high in resilience are less likely to feel entrapment. However, these moderation effects were not found for the entrapment‐to‐suicidal ideation pathway.

The study provided clear support for the temporal mediating role of entrapment between defeat and suicidal ideation, consistent with previous research (e.g., Wetherall, Cleare, Eschle, et al. 2022). By testing temporal mediation over a short time period, these findings extend previous cross‐sectional studies, showing that entrapment is strongly associated with suicidal ideation (Wetherall et al. 2018; Clement et al. 2023), and two‐wave prospective studies showing that entrapment prospectively predicts the relationship between defeat and suicidal ideation (Wetherall, Cleare, Eschle, et al. 2022; Branley‐Bell et al. 2019; Owen et al. 2018). The current study more strongly supports the causality hypothesis of the motivational phase as the mediation analysis was drawn from three time points, supporting the temporality assumed by mediation analysis (Narita et al. 2025). The analysis was also strengthened by the inclusion of baseline measures of the key variables, as this accounts for some of the potential confounding effects found within mediation analysis.

Sensitivity analyses further indicated that internal, but not external, entrapment mediated the longitudinal association between defeat and suicidal ideation. This aligns with prior research suggesting that internal entrapment, which reflects feelings of being trapped by one's own thoughts, emotions, or internal states, may be particularly pernicious for suicide risk compared to external factors, presenting a more proximal psychological mechanism linking defeat to suicidal thinking (Wetherall, Cleare, McClelland, et al. 2022; Owen et al. 2018; Höller et al. 2022). From a theoretical perspective, this finding supports the centrality of internally experienced escape motivation within the motivational phase of the IMV model, suggesting that it appears to be the internalization of defeating or humiliating experiences—manifesting as a sense of inescapable internal pain or cognitive–affective confinement—that most strongly drives the emergence of suicidal ideation (O'Connor and Portzky 2018).

The current findings suggest that when individuals experience persistent feelings of defeat and subsequent sense of entrapment, they may begin to consider suicide as a solution to this intolerable internal pain and external stress (Schuck et al. 2019). Individual and situational factors may lead to an individual experiencing feelings of defeat; these may include personality factors that convey vulnerability, such as perfectionism (Moscardini et al. 2023), or life events and stresses, such as job loss (van Eersel et al. 2021). Indeed, during the COVID‐19 pandemic there was some evidence that defeat may have been elevated at the start of the pandemic and then decreased over time (O'Connor et al. 2021), this may have been potentially exacerbated by the sense of uncertainty and lack of control that existed during this period. Further, being instructed not to leave your home could contribute to feelings of entrapment, and there is evidence that entrapment was elevated during lockdown periods (Wetherall, Cleare, McClelland, et al. 2022). However, the present findings are likely applicable beyond the pandemic context, and, although conducted during this period, the current analysis was not designed to model changes in mental wellbeing across the COVID‐19 pandemic.

The findings also highlight a key role for loneliness, suggesting that as levels of loneliness increase, the effect of defeat on entrapment also increases, thereby supporting previous research (McClelland et al. 2021). Moreover, others have noted that loneliness shares some similarities with other Threat to Self‐Moderators (TSM) in the motivational phase of the IMV model (e.g., rumination, coping, and social problem solving), and that although there are differences in these elements, they are all related to loneliness (Ernst et al. 2022; Chang et al. 2020). As a TSM that amplifies the transition from defeat to entrapment by undermining perceived social belonging and increasing negative self‐evaluations and sensitivity to perceived relational threat, the observed effects of loneliness also fit with the sociometer theory (Leary 2012), according to which loneliness signals low relational value and social exclusion, both of which likely intensify the psychological impact of defeat.

Previous research has found loneliness can also act as a Motivational Moderator (MM), strengthening the pathway from entrapment to suicidal ideation (McClelland et al. 2023). However, in the present study, no significant evidence of a moderating effect of loneliness on this pathway was found. Although it is conceptually close to motivational moderators such as thwarted belongingness, it also differs as it is typically unidimensional (i.e., a sense of disconnection) rather than the broader relational or interpersonal constructs that characterize other MMs (O'Connor and Kirtley 2018). However, due to these conflicting findings, future research should further explore different operationalizations of loneliness in the context of the IMV model. Importantly, loneliness can be reduced through targeted psychological interventions, including cognitive‐behavioral, social‐cognitive, mindfulness‐based, and digitally delivered programs, with meta‐analytic evidence demonstrating small to moderate effects across randomized trials (Hickin et al. 2021).

Interventions focused on resilience may also be fruitful. This is perhaps unsurprising, as resilience has been suggested to be critical for dealing effectively with challenges, uncertainty, and changes by moderating the relationship between risk factors such as defeat, entrapment, and despair (Gooding et al. 2015). Therefore, when individuals experience defeat in stressful situations, timely interventions for resilience may help to reduce feelings of entrapment and thus prevent the development of suicidal ideation. Although previous research has shown that resilience can moderate the relationship between entrapment and suicidal ideation (Li et al. 2021; Wetherall et al. 2018), this study did not support this moderating effect, which was not consistent with the IMV model. This may reflect the unpredictability of the early phase of the pandemic, with unprecedented levels of uncertainty meaning that protective factors may not have had their typical buffering effect (Nikopoulou et al. 2022), although this was not studied here. Evidence suggests that resilience training interventions have been effective in trials to reduce suicidal thoughts, with changes in resilience mediating the intervention's effects on changes in suicidal ideation (Zhang et al. 2022). Further, the literature suggests resilience can be a process (where people bounce back after adversity) or a characteristic that comprises personal and social resources (Ayed et al. 2019), highlighting that internal and external resources can enhance resilience in the protection of mental health and suicide risk (Egan et al. 2024).

The current study has several important implications. The impact of defeat and entrapment should be considered when developing interventions for individuals presenting with suicidal ideation. For those with self‐reported feelings of defeat, both their levels of loneliness and entrapment should be assessed when formulating intervention strategies; for individuals who do not report feelings of defeat, they may benefit from exploring feelings of loneliness (McClelland et al. 2021). Understanding the pathways to suicidal ideation could help inform more effective treatment strategies and reduce the risk of recurrence. In addition, this study found that resilience buffers the effect of defeat on entrapment. This implies that attention should be paid to enhancing resilience. Indeed, many studies have shown that emotional connection with family and friends and social support are associated with greater resilience (Killgore et al. 2020).

## 5 Limitations and Future Research

There are several limitations in this study. Firstly, all measures were derived from self‐report and therefore may be subject to reporting bias. Consistent with other non‐clinical suicide studies, the single measure of suicidal ideation may lead to errors in the data (Mars et al. 2016). Therefore, future research could combine the use of multiple data sources, such as self‐reported data, ecological transient assessments, and clinical assessments, combining quantitative and qualitative methods to provide a comprehensive measure of key psychological factors such as suicidal ideation (Türk et al. 2024).

Secondly, although a national sample was recruited based on quotas, the participants were mainly white (90.5%), so the findings do not reflect the applicability of the IMV model across different cultures and ethnicities. Like other studies that have recruited samples online, there may be a lack of representation of populations excluded by digital means. Participants who did not complete all waves of the survey tended to have poorer mental health (O'Connor et al. 2021), so the findings may not be representative of at‐risk populations in the sample. Thirdly, as this study was conducted during COVID‐19, it would be important to replicate these moderated mediation findings in other longitudinal contexts and to also explore these relationships in the longer term. Lastly, although our study provides a temporal test of the defeat–entrapment pathway central to the IMV model, we acknowledge that suicidal ideation can emerge from multiple psychological processes. Negative life events may evoke other proximal mechanisms, such as hopelessness, psychological pain, or psychological strain (e.g., Klonsky and May 2015; Lyu et al. 2020), that are not captured in our models. The IMV model does not posit that defeat and entrapment constitute the sole pathway, and future research should compare the temporal strength of these mechanisms against alternative theoretical frameworks.

## 6 Conclusion

This study tested key premises of the motivational phase of the IMV model using temporal variables and controlling for baseline confounders. The study provides robust evidence of the causality of the variables within the motivational phase of the IMV model by demonstrating that entrapment plays a longitudinal mediating role in the pathway from defeat to suicidal ideation. Loneliness and resilience moderated the pathway from defeat to entrapment, respectively, and may be key targets for intervention. However, there was no evidence to support a moderating effect of loneliness and resilience on the pathway from entrapment to suicidal ideation. The findings highlight the influence defeat and entrapment exert upon the emergence of suicidal ideation.

## Author Contributions

**Jiarui Xiao:** investigation (lead), formal analysis (lead), writing – original draft (lead). **Karen Wetherall:** conceptualization (supporting), methodology (equal), data curation (equal), investigation (equal), formal analysis (supporting), supervision (lead), project administration (equal), writing – review and editing (lead). **Seonaid Cleare:** conceptualization (supporting), methodology (equal), data curation (equal), project administration (equal), writing – review and editing (equal). **Mareike Ernst:** investigation (supporting), formal analysis (supporting), writing – review and editing (equal). **Kathryn A. Robb:** conceptualization (equal), methodology (equal), investigation (equal), funding acquisition (equal), writing – review and editing (equal). **Rory C. O'Connor:** conceptualization (lead), methodology (equal), investigation (equal), supervision (supporting), funding acquisition (lead), writing – review and editing (equal).

## Ethics Statement

Ethical approval was obtained from the University of Glasgow's Medical, Veterinary, and Life Sciences Ethics Committee (approval number: 200190146) and the study was conducted in accordance with the Declaration of Helsinki (World Medical Association 2001). Participants provided informed consent prior to participating and could withdraw at any time.

## Conflicts of Interest

The authors declare no conflicts of interest.

## Supporting information


**Figure S1:** Model 1: mediation model with entrapment mediating the relationship defeat and suicidal ideation (*n* = 2401).
**Table S1:** Table of each moderator model output with internal entrapment as the mediator (model, moderator and outcome variable specified).
**Figure S2:** Moderating effects of loneliness on the pathway from entrapment to suicidal ideation (*n* = 2401).
**Figure S3:** Moderating effects of resilience on the pathway from entrapment to suicidal ideation (*n* = 2401).

## Data Availability

Anonymized data variables used within this study can be accessed through the Open Science Framework (https://osf.io/574dv/files/osfstorage).
