# Supplementary material for: Longitudinal Temporal Mediation Within the Motivational Phase of the Integrated Motivational‐Volitional (IMV) Model of Suicidal Behavior With Moderation of Loneliness and Resilience
Source: Suicide Life Threat Behav. 2026 Feb 22;56(1):e70081. doi: 10.1111/sltb.70081 (PMC12926524; doi:10.1111/sltb.70081)
Supplement: Supplementary file 1 — Figure S1: Model 1: mediation model with entrapment mediating the relationship defeat and suicidal ideation (n = 2401). Table S1: Table of each moderator model output with internal entrapment as the mediator (model, moderator and outcome variable specified). Figure S2: Moderating effects of loneliness on the pathway from entrapment to suicidal ideation (n = 2401). Figure S3: Moderating effects of resilience on the pathway from entrapment to suicidal ideation (n = 2401). [file SLTB-56-0-s001.docx]

**Supplementary Materials**

1. **Internal and external entrapment as parallel mediators of the defeat to suicidal ideation pathway (Model 2) and moderated mediation findings with internal entrapment as mediator**

**Figure S1.** **Model 1: mediation model with entrapment mediating the relationship defeat and suicidal ideation (n=2401).**

**External entrapment (Wave 2)**

0.158***

**Defeat (Wave 1)**

**Suicidal ideation (Wave 3)**

**Internal entrapment (Wave 2)**

0.137^***^

0.279^***^

0.026

0.091

^*^p<0.05, ^**^p<0.01, ^***^p<0.001. Analysis controlling for entrapment and suicidal ideation in Wave 1. Indirect effect of internal entrapment: β = 0.037, S.E. = 0.011, CI [0.018, 0.059], external entrapment: β = 0.014, S.E. = 0.011, CI [-0.008, 0.037].

**Table S1: Table of each moderator model output with internal entrapment as the mediator (model, moderator and outcome variable specified).**

| Model and moderator: | Model 3: Loneliness wave 1 | Model 4: Loneliness wave 2 | Model 5: Resilience wave 1 | Model 6: Resilience wave 2 |
| --- | --- | --- | --- | --- |
| Outcome: | Internal entrapment  b [95%CI] | Suicidal Ideation  b [95%CI] | Internal entrapment  b [95%CI] | Suicidal ideation  b [95%CI] |
| Wave 1 defeat | .036 [-.011, .083] | -.010 [-.081, .061] | **.209 [.156, .260]** | -.019 [-.090, .052] |
| Wave 1 internal entrapment | **.489 [.448, .530]** | .055 [-.061, .171] | .**503 [.462, .544]** | .061 [-.056, .178] |
| Wave 1 suicidal ideation | **.390 [.163, .618]** | **2.719 [2.301, 3.137]** | **.435 [.207, .663]** | **2.726 [2.306, 3.146]** |
| Moderator | .040 [-.006, .086] | .085 [-.063, .233] | **-.022 [-.043, -.001]** | -.067 [-.139, .005] |
| Wave 2 internal entrapment | _ | **.401 [.165, .638]** | _ | **.259 [.046, .471]** |
| Interaction | **.018 [.010, 0.025]** | -.009 [-.042, .025] | **-.005 [-.009, -.002]** | .002 [-.013, .018] |

Note: 95% CI not crossing zero indicates significant finding (in bold).

1. **Moderating effects of loneliness on the pathway from entrapment to suicidal ideation (Model 4)**

In this study, we tested whether loneliness moderated the pathway from entrapment to suicidal ideation. As shown in Figure S2, entrapment significantly predicted suicidal ideation (β = 0.204, S.E. = 0.066, CI [0.075, 0.333], p = 0.002), indicating that an increase in entrapment leads to an increase in suicidal ideation. However, moderation analyses revealed that loneliness did not significantly moderate the pathway from entrapment to suicidal ideation (β = -0.004, S.E. = 0.009, CI [-0.022, 0.015], p = 0.696), suggesting that level of loneliness did not significantly influence the predictive effect of entrapment on suicidal ideation.

**Loneliness (Wave 2)**

**Defeat (Wave 1)**

**Suicidal ideation (Wave 3)**

**Entrapment (Wave 2)**

0.014^***^

0.204^**^

0.019

-0.004

**Figure S2** Moderating effects of loneliness on the pathway from entrapment to suicidal ideation (n=2401).

^*^p<0.05, ^**^p<0.01, ^***^p<0.001. Analysis controlling for entrapment and suicidal ideation in Wave 1.

1. **Moderating effects of resilience on the pathway from entrapment to suicidal ideation (Model 6)**

We investigated the potential moderating effect of resilience on the relationship between entrapment and suicidal ideation. As shown in Figure S3, there was a significant positive relationship between entrapment and suicidal ideation (β = 0.160, S.E. = 0.059, CI [0.044, 0.276], p = 0.007), demonstrating that higher levels of entrapment are associated with increased suicidal ideation. However, the moderation analysis showed that resilience did not significantly alter the impact of entrapment on suicidal ideation (β = 0.001, S.E. = 0.004, CI [-0.007, 0.009], p = 0.798), indicating that resilience levels did not significantly affect how entrapment predicts suicidal ideation.

**Resilience (Wave 2)**

**Defeat (Wave 1)**

**Suicidal ideation (Wave 3)**

**Entrapment (Wave 2)**

0.378^***^

0.160^**^

0.009

0.001

**Figure S3** Moderating effects of resilience on the pathway from entrapment to suicidal ideation (n=2401).

^*^p<0.05, ^**^p<0.01, ^***^p<0.001. Analysis controlling for entrapment and suicidal ideation in Wave 1.
